# LDMD: A database of microbes in human lung disease

**DOI:** 10.3389/fmicb.2022.1085079

**Published:** 2023-01-10

**Authors:** Li-Qun Xu, Jing Yang, Weicheng Liang, Jiang Chen, Zepeng Sun, Qiang Zhang, Xinlong Liu, Feng Qiao, Jian Li

**Affiliations:** ^1^China Mobile (Chengdu) Industrial Research Institute, Chengdu, China; ^2^Key Laboratory of DGHD, MOE, School of Life Science and Technology, Southeast University, Nanjing, China; ^3^Department of Respirology, Zhongda Hospital, Southeast University, Nanjing, China

**Keywords:** lung disease, blast, database, microorganisms, metagenome

## Abstract

**Background:**

Lungs were initially thought to be sterile. However, with the development of sequencing technologies, various commensal microorganisms, especially bacteria, have been observed in the lungs of healthy humans. Several studies have also linked lung microbes to infectious lung diseases. However, few databases have focused on the metagenomics of lungs to provide microbial compositions and corresponding metadata information. Such a database would be handy for researching and treating lung diseases.

**Methods:**

To provide researchers with a preliminary understanding of lung microbes and their research methods, the LDMD collated nearly 10,000 studies in the literature covering over 30 diseases, gathered basic information such as the sources of lung microbe samples, sequencing methods, and processing software, as well as analyzed the metagenomic sequencing characteristics of lung microbes. Besides, the LDMD also contained data collected in our laboratory.

**Results:**

In this study, we established the Lung Disease Microorganisms Database (LDMD), a comprehensive database of microbes involved in lung disease. The LDMD offered sequence analysis capabilities, allowing users to upload their sequencing results, align them with the data collated in the database, and visually analyze the results.

**Conclusion:**

In conclusion, the LDMD possesses various functionalities that provide a convenient and comprehensive resource to study the lung metagenome and treat lung diseases.

## 1. Introduction

According to current research statistics, the human body contains roughly the same number of cells and bacteria (Sender et al., 2016). Over the past decade, research on the human microbiome has expanded from other microbe-rich environments, such as the gut, to organs that were previously considered sterile, such as the bladder and lungs. Although the lungs are known to contain microorganisms in acute infections and chronic suppurative diseases, recent culture-independent methods have described microbial communities in healthy lungs (Hilty et al., 2010; Erb-Downward et al., 2011; Melo-Dias et al., 2022). Previously, lungs were widely considered sterile due to some misinterpretation of concepts and data. For example, failure to culture microorganisms from airway samples using growth conditions established for the detection of known pathogens has been interpreted as the absence of microorganisms rather than a technical limitation due to culture conditions (Dickson et al., 2016). Given their proximity to the microbe-rich upper respiratory tract, it is not surprising that the lungs are not sterile. However, healthy lungs have very low microbial biomass, with 10^3^–10^5^ bacteria per gram of tissue (Mathieu et al., 2018) (by comparison, the large intestine has a density of 10^11^–10^12^ bacteria per gram), and the nature of the lung microbiota is significantly different in terms of quantity and kinetics compared to other body habitats where the microbiota flourish, such as the gut, skin, mouth, and vagina (Whiteside et al., 2021). An increasing number of studies have demonstrated that the lungs of healthy people contain a microbiome (Hilty et al., 2010; Erb-Downward et al., 2011; Morris et al., 2013; Bassis et al., 2015; Segal et al., 2016; Yu et al., 2016; Pattaroni et al., 2018), the main genera of which are *Prevotella*, *Streptococcus*, *Veillonella*, *Clostridium*, and *Hemophilus* (Dickson et al., 2016; Hooks and O'Malley, 2017; Huffnagle et al., 2017). Unfortunately, a specific database focusing on lung microbes and their correlation with lung diseases is still lacking, limiting the application of advanced sequencing technologies in the diagnosis and treatment of diseases.

Several respiratory diseases are now thought to be associated with dysregulated proportions of pulmonary microbiota, although research is only at the initial stage of addressing causality and underlying mechanisms (Hooks and O’Malley, 2017; Levy et al., 2017). Therefore, the study of pulmonary microbiota has attracted several research activities in recent years. The wide use of next-generation sequencing technologies has provided the means to revolutionize research on microbiota, and data on lung diseases and microbial sequences has witnessed tremendous growth, indicating that research on the impact of lung microbiota on respiratory health has entered a new era. However, almost all studies on the lung microbiome until now have been observational. Although studies on the lung microbiome have confirmed that the microbial composition in lung diseases such as asthma and chronic obstructive pulmonary disease differs from that in healthy subjects, the description of the lung microbiome alone is insufficient to provide an understanding of the mechanisms. Moreover, there are no strict normative guidelines for microbiome research design, experiments, detection methods (16S rRNA gene sequencing, shotgun metagenomics gene sequencing, and whole genome sequencing), and microbiome data analysis (Carney et al., 2020; Yagi et al., 2021).

In contrast to traditional culture methods, which focus on studying individual organisms, microbiome research uses a sequence-based approach to document the entire community. The most widely used method uses PCR to amplify and sequence a region shared by all members of the group, such as the 16S rRNA gene of bacteria and the 18S rRNA gene of fungi, and then determine its population (Carney et al., 2020). However, full-length 16S rRNA gene sequencing has limited application in microbial sequencing of the lungs (Toma et al., 2014; Wang et al., 2020), whereas short-read long 16S rRNA gene-variable region sequencing is the most common research method. Another major research method is shotgun metagenomic sequencing, which sequences all the DNA in a sample. Although the presence of a high percentage of host-derived DNA in lung samples is a challenge, shotgun sequencing is increasingly being used in lung studies (Millares et al., 2015; Cameron et al., 2017; Marotz et al., 2018; Mac Aogáin et al., 2020; Sulaiman et al., 2021). The interpretation of 16S rRNA gene sequencing and shotgun sequencing is related to the corresponding reference database; due to the possible low abundance of taxa, it will be difficult to distinguish target sequences from background pollutant sequences (Kaakoush, 2015; Thomas and Segata, 2019). However, the complexity of lung microbial sequencing data and the rapid increase in its amount pose a major challenge to using lung disease microbial data. Current studies have shown that the collection of datasets of lung diseases and related microorganisms plays a practical role in the development of treatment strategies against lung diseases, which supports the necessity for the establishment of a microbial database for lung diseases (Yi et al., 2021).

With the rapid development of high-throughput metagenomic sequencing technology, various human microbial sequencing data, including 16S amplicon sequencing profiles and microbial whole genome sequencing profiles, have been collected. Several pioneering studies have been conducted to construct resources that store raw sequencing data, such as the Sequence Read Archive (SRA) of the National Center for Biotechnology Information (NCBI; Kodama et al., 2012), the European Nucleotide Archive (ENA; Harrison et al., 2019), and the Japanese DNA Database (DDBJ; Mashima et al., 2017). Among the established databases, the NCBI database is a comprehensive database that is widely used in biology. It stores data from the genome, transcriptome, and proteome and has remarkably contributed to the development of relevant research (Sayers et al., 2021). However, it has some limitations. First, because of the wide range of data collected, it contains a considerable amount of non-pulmonary microbiological data. However, there is a relative scarcity of data on the microbiome of lung diseases (e.g., literature on the relationship between the microbiome and lung disease and other publicly available data). Moreover, although some public databases provide comprehensive data collection, there is a lack of correlation between standardized methods and management systems for specific multidimensional microbial data and pulmonary disease data. On the other hand, the database Disbiome (Janssens et al., 2018) collects and displays published information on the microbial disease in a standardized manner, MicrobiomeDB (Oliveira et al., 2018) (a data discovery and analysis platform) enables researchers to fully utilize experimental variables to query microbiome datasets and mBodyMap (Jin et al., 2022) identifies the microorganisms in the human body and establishes their relationship with health and diseases, which helps identify the pathogenic microorganisms.

Although these databases provide an important resource for the study of the microbiome, they either focus on multi-site diseases or tend to annotate a wide range of microorganisms and do not exhibit microbial changes in different lung diseases. Given the urgent need for a specific dataset for lung microbes, we created this Lung Disease Microbiome Database (LDMD) to prepare for future research and provide a comprehensive and searchable database for scholars in related fields. Also, to facilitate the use of this database, we implemented the browsing and searching functions and carefully organized the information in the database. By establishing relationships between lung diseases and microbes, the LDMD, unlike existing databases, allows researchers to obtain microbes for lung diseases that they are interested in or compare their results with published studies.

## 2. Materials and methods

### 2.1. Collection of raw data

The design of standardized data collection and organization process is necessary to improve the quality of LDMD created in this study. Keyword combinations such as “lung disease and sequencing,” “lung disease and microbes,” “lung disease and 16S,” and “lung disease and metagenome” were selected, and relevant works in literature were retrieved from PubMed (Fiorini et al., 2017). Based on the abstract and the complete text, we manually filtered the data to extract the methods used in the literature for microbial sequencing analysis and the representative microorganisms of lung diseases to facilitate the preliminary understanding of the general situation among researchers and the research methods for lung microorganisms. A total of 10,000 relevant works were collected from the literature, and necessary microbial information was obtained from the collected literature for further processing. At the same time, relevant experimental information, such as the sample source, sequencing method, and processing software, was also collected.

### 2.2. Data processing

Because only a part of the data (obtained from the literature) was relatively complete, we obtained information on diseases, related microorganisms, value of p, and processing software from the literature using data classification, thus creating a table for induction and sorting. On the other hand, data that were not available in the literature were considered missing. Similarly, we also filtered out data from over 3,000 microbes from lung diseases according to the kingdom, phylum, class, order, family, genus, and species to classify the microbes.

### 2.3. Taxon rematch

**Figure 1:**
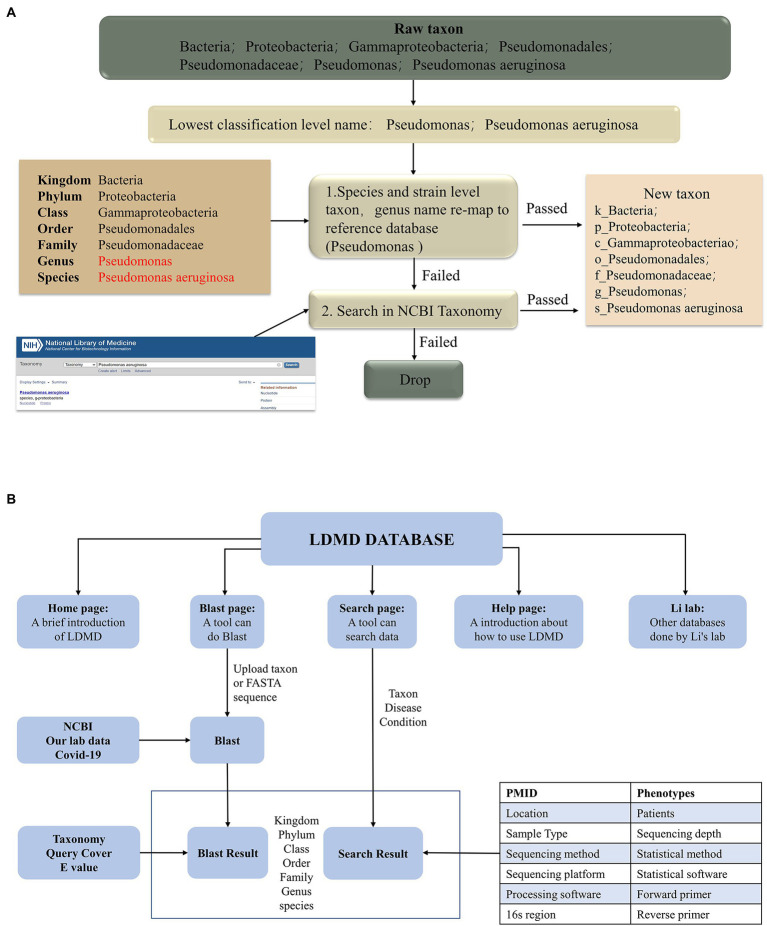
The workflow for creating the LDMD. **(A)** The process of taxon rematch. **(B)** The main framework of LDMD.

As different microbial reference databases can lead to taxonomic conflicts, it becomes necessary to collate the names of microorganisms for consistent taxonomic identification. Figure 1A shows the LDMD rematch process, where we mapped each taxon to a detailed classification level using the integrated reference database. All taxa were remapped to full taxon names in three steps. First, the originally collected taxa were consolidated on the lowest taxonomic level names, which were then remapped to the NCBI classification criteria to obtain their new taxa. Second, in the remaining taxa, we remapped the genus names to the NCBI classification at the species or strain level and retained them when successful. Finally, we searched for the remaining taxa in the NCBI Taxonomy to see if any of them had new names, and the taxa that were not verified by the database were deleted.

### 2.4. Implementation of a web server

**Figure 2:**
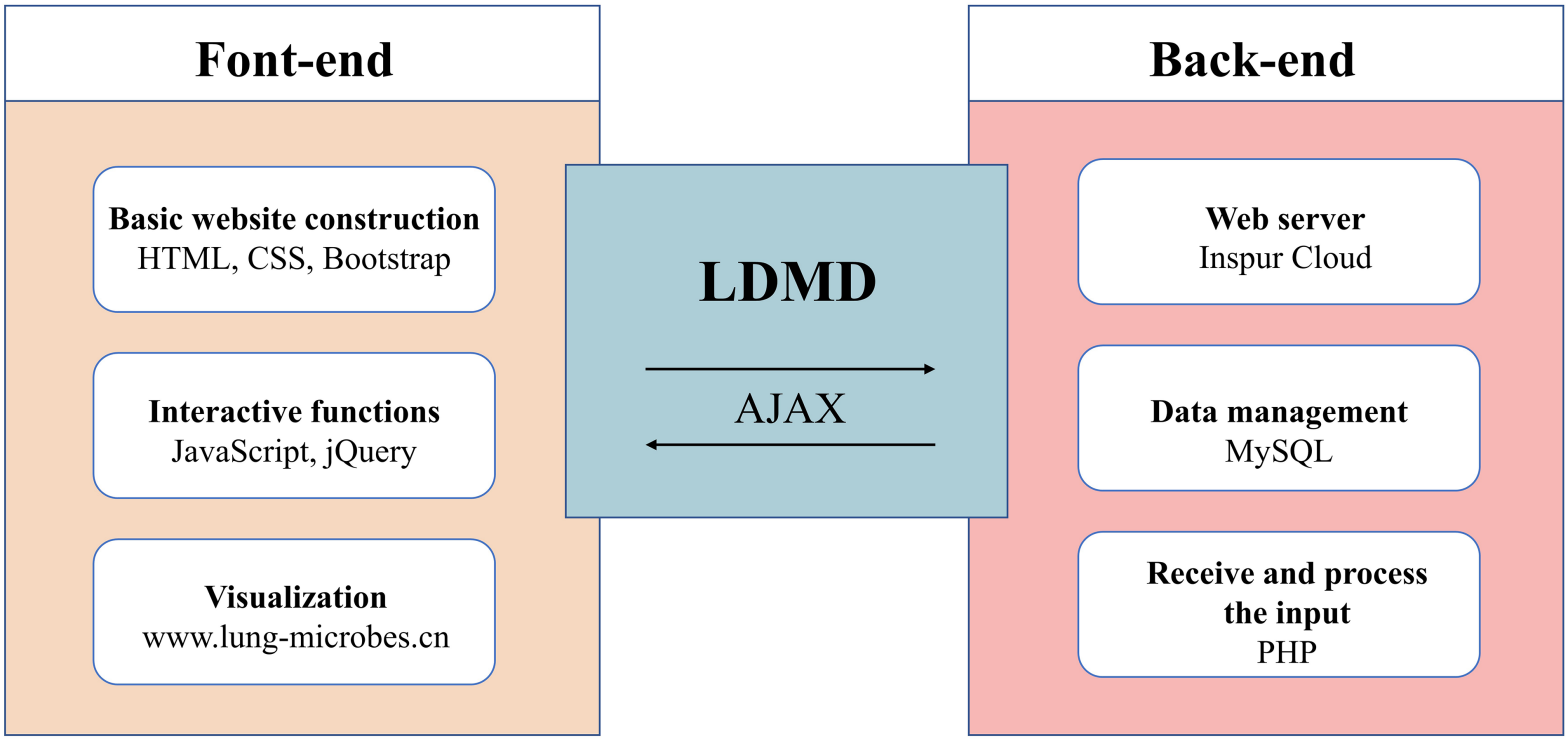
The main architecture of the LDMD website.

The data were collected and stored in the MySQL database, while the website was built using HTML, JavaScript, and PHP. The website was hosted on the Inspur Cloud server. Moreover, to provide a robust service, we tested the LDMD website on various web browsers such as Mozilla Firefox, Google Chrome, and Microsoft Edge (Supplementary Figures S1–S3 of supplementary materials). Figure 2 describes the basic structure of the LDMD database.

## 3. Results

### 3.1. The workflow and composition of LDMD

We collected a total of 134 studies and further processed the relevant data so that all taxa were remapped to the full classification (Figure 1A). The LDMD was divided into several components, including the home page, BLAST comparison, search query, operation help, and relevant website navigation. The main framework, interaction, and logic of LDMD are presented in Figure 1B.

**Figure 3:**
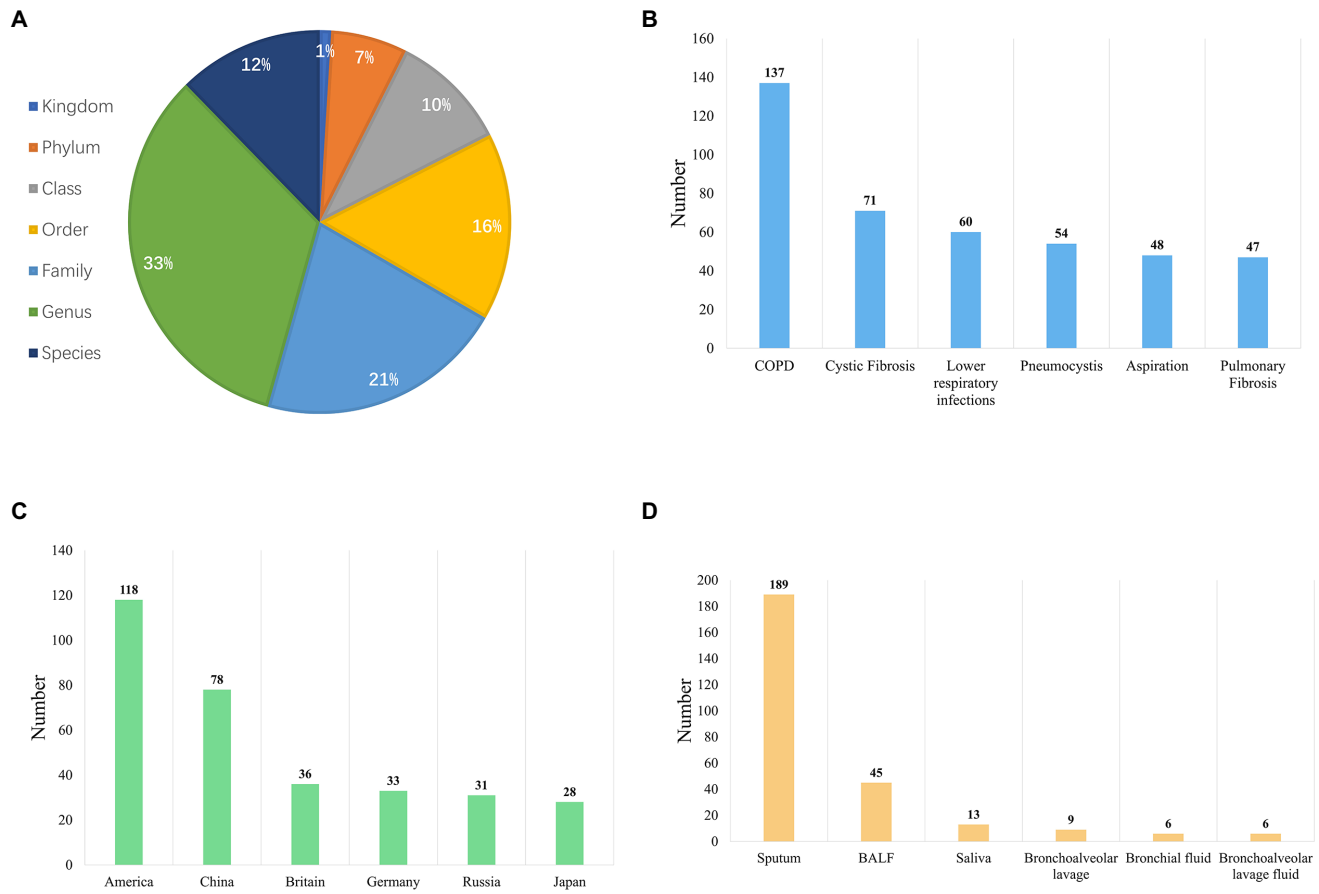
Overview of the data composition in LDMA. **(A)** The distribution of quantitative events in different taxonomic levels (kingdom, phylum, class, order, family, genus, and species). **(B)** The distribution of quantitative events in different conditions (top 6). **(C)** The distribution of human lung metagenomic research in different countries (top 6). **(D)** The types of samples used for DNA extraction are based on our literature review.

In the current implementation, LDMD integrates seven taxonomic levels of quantified events under different conditions (Figure 3A), distributed in bounds (3, 1%), phyla (20, 7%), classes (31, 10%), orders (49, 16%), families (65, 21%), genera (103, 33%), and species (38, 12%). Among these taxonomic levels, genera and species have been extensively studied. We also observed that 16S amplicon sequencing could distinguish microbiota into genera, whereas whole-genome sequencing can discern species, which was consistent with the collected data (Breitwieser et al., 2019). Moreover, the top six diseases included chronic obstructive pulmonary disease (COPD), cystic fibrosis, lower respiratory cell infections, pneumocystis, aspiration, and pulmonary fibrosis (Figure 3B). A large proportion of the collected study sample data was from the United States (Figure 3C). Sputum was the main source of microbial DNA, followed by BALF and saliva, among others (Figure 3D). The literature revealed that sequencing was mainly performed using the platform Illumina MiSeq, with the 16S amplicon being targeted for sequencing. The Mann–Whitney *U*-test was used to statistically compare data between groups (Supplementary Figure S4 supplementary materials). Thus, the LDMD contained a large amount of laboratory-generated data collected from the literature, which complemented public data and formed the data source for LDMD.

### 3.2. Query function and result presentation for LDMD

**Figure 4:**
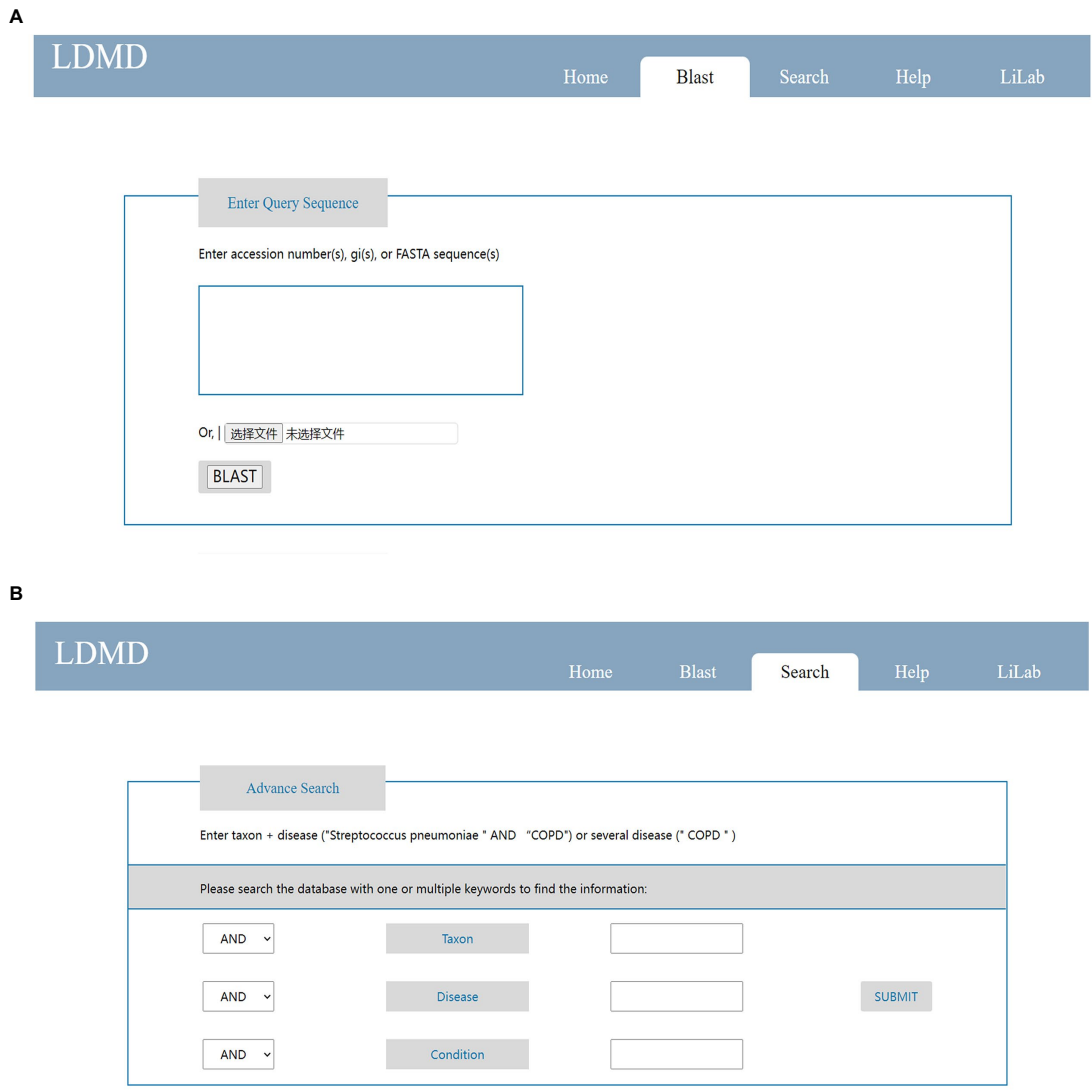
Main pages on LDMD to query results. **(A)** The simple search function on the home page. **(B)** The advanced search function on the search page.

The LDMD database provided researchers with two query modes to obtain genomic information about lung microorganisms of interest in a user-friendly manner. First, LDMD provided a BLAST search to query the database. Users could launch a fast retrieval on the home page by submitting data in FASTA format (Figure 4A). Second, we provided “Advanced Search” capabilities on the search page so that users could enter multiple keywords and get more accurate results (Figure 4B).

**Figure 5:**
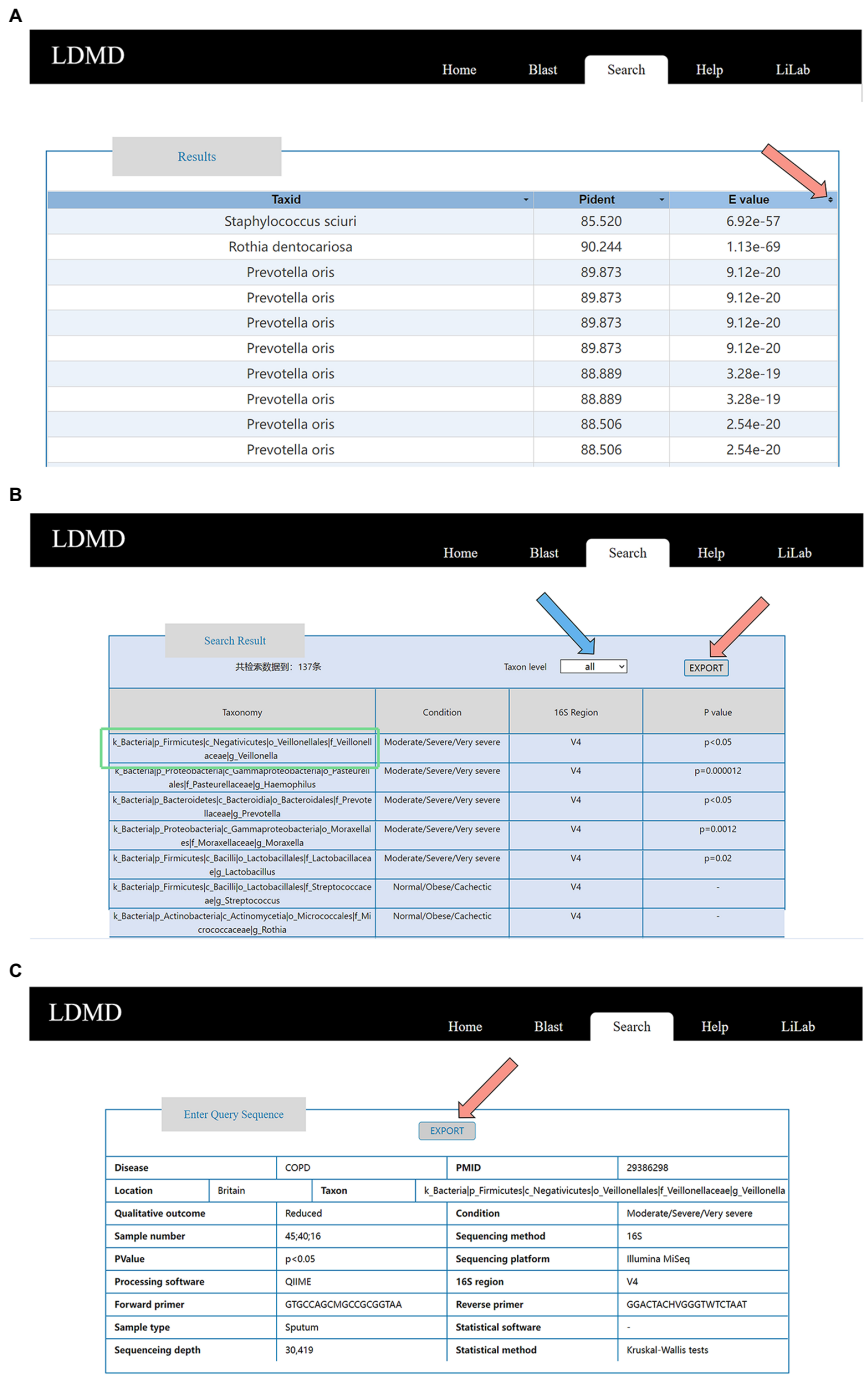
Detailed information for query results. **(A)** Result table on the BLAST result page. **(B)** Result table on the Advanced result page. **(C)** Experimental information, including PMID, location, sample type, number of participants, phenotypes, sequencing method and platform, statistical method and software, main processing software, 16S region, forward primer, and reverse primer for the amplification of the 16S region.

Each query generated a corresponding result. When we uploaded a FASTA file and compared it with BLAST, the BLAST retrieval results included Taxid, Pident, and E-value (Figure 5A). Users could view specific results by clicking the icon on the E-value (pointed to by the arrow in Figure 5A). After using COPD as the keyword of disease as an advanced search, the results could be divided into taxonomy, condition, 16S region, and value of p (this value of p indicates if the taxon is associated with the disease). Researchers could click the taxon level button pointed to by the blue arrow to classify the results. They could also click the EXPORT button pointed to by the red arrow to export the retrieval results (Figure 5B). Moreover, researchers could obtain experimental information, such as sample type, sequencing platform, and statistical software, by clicking the results in “Taxonomy” (the content in the green box). This experimental information can then be exported by clicking the EXPORT button (Figure 5C). This exported information is stored locally in Microsoft Excel format. If the data are from 16S amplicon sequencing, further information about the amplified regions and primers is provided. In general, associated queries can be made to identify specific associations between lung diseases and microorganisms. Users can also obtain the corresponding microbial data to provide a specific reference through this function. This will further facilitate data exchange and sharing of lung disease microorganisms, which is essential to accelerate research on lung disease and promote the understanding of the association between lung diseases and microorganisms.

### 3.3. Example of LDMD usage

**Figure 6:**
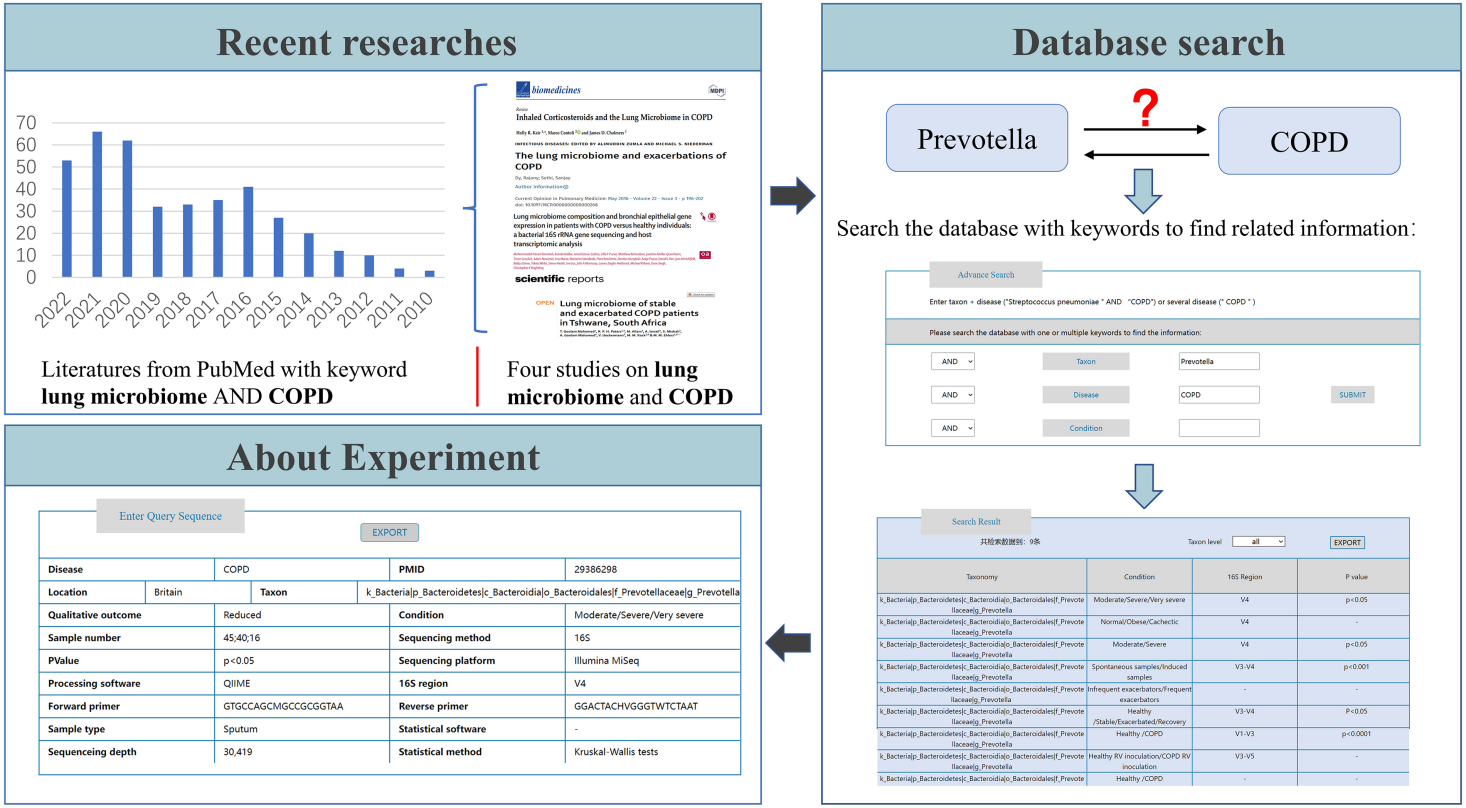
An example of LDMD usage.

To better understand the usage of LDMD, we provide an example in Figure 6. Studies have indicated that lung microbes may contribute to COPD (Dy and Sethi, 2016; Goolam Mahomed et al., 2021; Keir et al., 2021; Ramsheh et al., 2021), and we were interested in whether *Prevotella* plays a role in the occurrence and development of COPD. Therefore, we needed to understand the variations in the abundance of *Prevotella* in COPD before starting the experiment to ensure the accuracy of the experimental design. To that end, the keywords “*Prevotella*” taxon and “COPD” disease were used to search the database. The search results indicated that a significant presence of *Prevotella* has been reported in COPD patients in the relevant literature, which further supported the association between *Prevotella* and COPD. This suggested that *Prevotella* could be involved in the occurrence and development of COPD. Based on these results, we could also learn more about the experimental information in LDMD, which helped in the subsequent experimental verification. If further information on the source of the data was required, we also provided the related paper PMID to users.

Some researchers have confirmed that *Prevotella* plays a role in the development of COPD (Mayhew et al., 2018), which proves that the use of LDMD plays an auxiliary role in scientific research. Therefore, if we want to discover, analyze, and verify associations between lung disease and bacteria like those between COPD and *Prevotella* through experiments, we can first use LDMD as a verification resource to consult existing studies and obtain corresponding experimental information to improve the accuracy of the experimental design. Overall, our database provides comprehensive information on disease-related microbial changes and serves as a resource for researchers to explore and refer to.

## 4. Discussion

With the rapid development of modern science and technology, the lung microbiome has attracted extensive attention. In contrast to the previously widely accepted theory that the lung is sterile, modern scientific studies have shown that the lung has a unique microbiome (Dickson et al., 2016; Wypych et al., 2019). Moreover, this theory has broad application prospects in studies on the pathogenesis of pulmonary disease and drug screening, as well as plays a key role in various fields, including life sciences and medicine. Lung microbes are essential to disease development and human health in diseases such as asthma (Huang et al., 2015; Simpson et al., 2016; Durack et al., 2017; Arrieta et al., 2018; Begley et al., 2018; Taylor et al., 2019) and COPD (Pragman et al., 2012; Wilkinson et al., 2017; Ghebre et al., 2018; Wang et al., 2019; Haldar et al., 2020). With continuing research on lung disease and associated microbes, a significant amount of data are generated. Several studies related to lung diseases and microbes continue to be published, and research on lung disease and microbiology is coming of age. Therefore, a database of lung diseases and microorganisms must be established. In this study, we not only introduced LDMD as a database of human lung diseases and microbes under different conditions but also created a user-friendly interface to accurately and intuitively display the results.

After our research, we observed that when clinical researchers studied the etiology of lung diseases, they always reviewed a large amount of literature for one disease, while in some cases, they even needed experimental verification. By creating LDMD, we could greatly facilitate the clinical workers. Firstly, researchers could save the time required for literature review and reduce the treatment cycle of patients by using LDMD. Secondly, they could obtain relevant experimental information through our search function, which reduced the resource loss of researcher-blind experiments. Finally, they could obtain the published research results quickly through our unique matching search. Therefore, LDMD could greatly help clinical researchers in research.

In this online LDMD sharing platform, we collected and sorted about 10,000 papers on over 30 diseases and further processed the relevant data for in-depth mining to screen out information, including the location, microorganism, and phenotype. The LDMD provided the lung microbiome research community with data sharing and application services, as well as different classification levels of lung microbial metagenome data. Therefore, users could intuitively obtain information about lung microorganisms under different conditions. Moreover, a comprehensive collection of microbial lung information related to diseases, such as COPD, cystic fibrosis, lower respiratory infections, pneumocystis, aspiration, and pulmonary fibrosis, was included so that LDMD could provide evidence of a link between microorganisms and the onset and progression of the disease. Similarly, our database contained two search pages: the BLAST page, which provides researchers with free online tools, such as BLAST sequence alignment, allowing them to upload and analyze their data or data of interest so that users could quickly and accurately obtain the required information. Also, the search page could be used for querying the association between lung diseases and microorganisms. This dual-search capability significantly facilitated the work of medical researchers. Combining these features, LDMD aimed to serve researchers interested in exploring the relationship between human health and microbial lung changes. Therefore, researchers could quickly obtain valuable references before or after the experiment, thus improving their work efficiency significantly.

Compared to other databases with a wide range of contents, LDMD mainly targets lung microorganisms, thus being highly pertinent and convenient, which can greatly save the time needed for comparison and facilitate the study of lung microorganisms by researchers in this field. Overall, the LDMD is a standardized system that contains several charts, literature, and sequencing data. Furthermore, the contents of the database are updated regularly. Literature and other relevant information are collected monthly to apprise researchers of the latest developments and research hotspots. Therefore, LDMD provides the most comprehensive, real-time, and dynamic progress in the microbiological research of lung diseases to researchers and health experts worldwide from industry and government agencies. In general, although various databases store metagenomic data, provide analytic processes, and even provide the microbial composition of samples and corresponding metadata, databases that display the dynamic changes in microbiota under different conditions need to be established. As changes in the lung microbiota are critical to human health in different temporal and spatial contexts, comprehensive human lung microbial genome resources can facilitate the reuse of published metagenomic datasets and greatly help metagenomic studies (Zhang et al., 2021). However, considerable work is still needed to establish a convenient and practical metagenomic data resource on lung microbes. First, all raw data can be re-analyzed on a well-established and standardized basis to provide more detailed and standardized quantitative information. In future studies, we aim to continuously improve the LDMD database and update it yearly. Although improvements are still needed, LDMD can be used as a comprehensive resource to provide intuitive data evidence for studies on lung microbiology. In summary, our database has facilitated advances in microbiology in lung disease. Moreover, due to the COVID-19 pandemic, we plan to increase the collection of relevant data on lung microbes to contribute to the ongoing fight against the epidemic.

## Data availability statement

Our original research and major findings are presented at http://www.lung-microbes.cn/, not in supplementary materials.

## Author contributions

L-QX and JL jointly led the overall project design, conceptualization, investigation, supervision, acquisition of funding and resources, and the manuscript review. JY performed the design of the database, collected the literature references and data, and participated in the manuscript drafting. WL collected the data and built the database. JC participated in building the database and drafted the manuscript. ZS, QZ, XL, and FQ carried out the overall project conceptualization, user requirements definition, and extensive tests. All authors contributed to the article and approved the submitted version.

## Funding

The authors are thankful for the support of the National Natural Science Foundation of China (32270607 and 31871322) and China Mobile (Chengdu) Industrial Research Institute (8531000015). The author also thanks for supporting the Space Medical Experiment Project of China Manned Space Program (HYZHXM01004).

## Conflict of interest

The authors declare that the research was conducted in the absence of any commercial or financial relationships that could be construed as a potential conflict of interest.

## Publisher’s note

All claims expressed in this article are solely those of the authors and do not necessarily represent those of their affiliated organizations, or those of the publisher, the editors and the reviewers. Any product that may be evaluated in this article, or claim that may be made by its manufacturer, is not guaranteed or endorsed by the publisher.
