# Supplementary material for: LDMD: A database of microbes in human lung disease
Source: Front Microbiol. 2023 Jan 10;13:1085079. doi: 10.3389/fmicb.2022.1085079 (PMC9873265; doi:10.3389/fmicb.2022.1085079)
Supplement: Supplementary file 1 [file Data_Sheet_1.PDF]

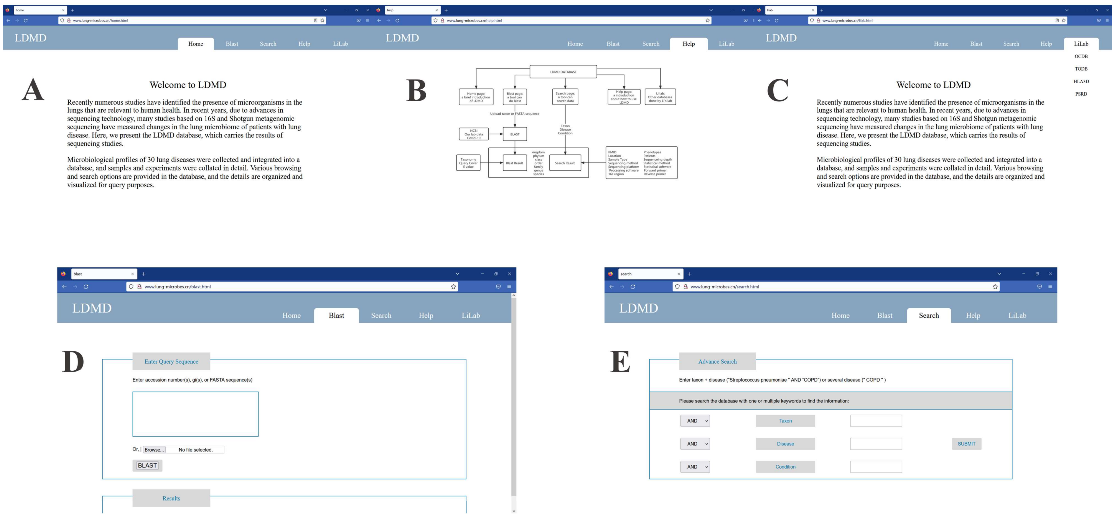

Figure S1 A-E

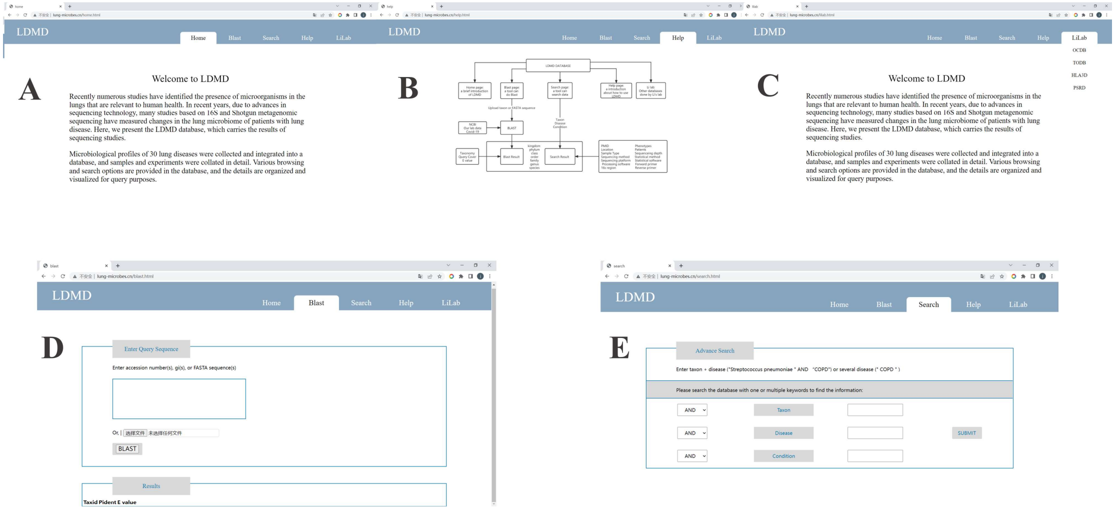

Figure S2 A-E

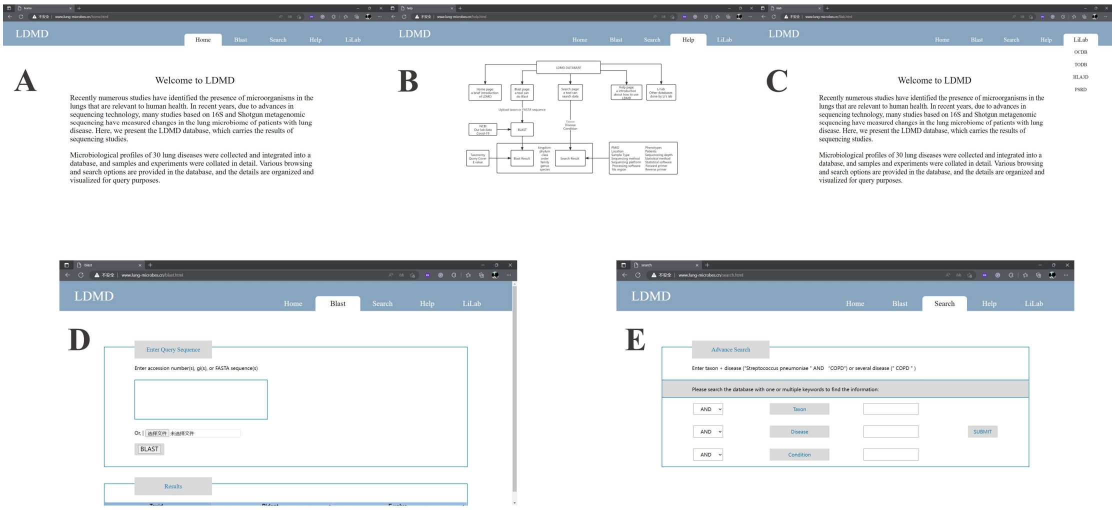

Figure S3 A-E

Figure S1 to Figure S3 respectively describe the testing of LDMD in Mozilla Firefox, Google Chrome, and Microsoft Edge. Figure A is the home page; Figure B is the help page, which describes the LDMD framework; Figure C is the website navigation of other databases in the laboratory; Figure D shows the Blast page; Figure E is the search page;

A

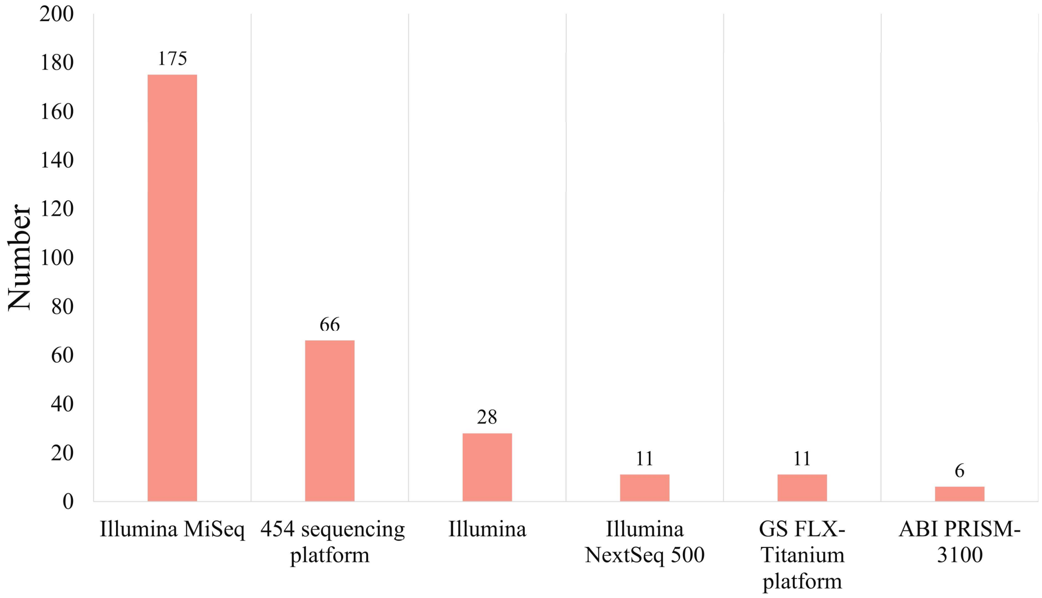

B

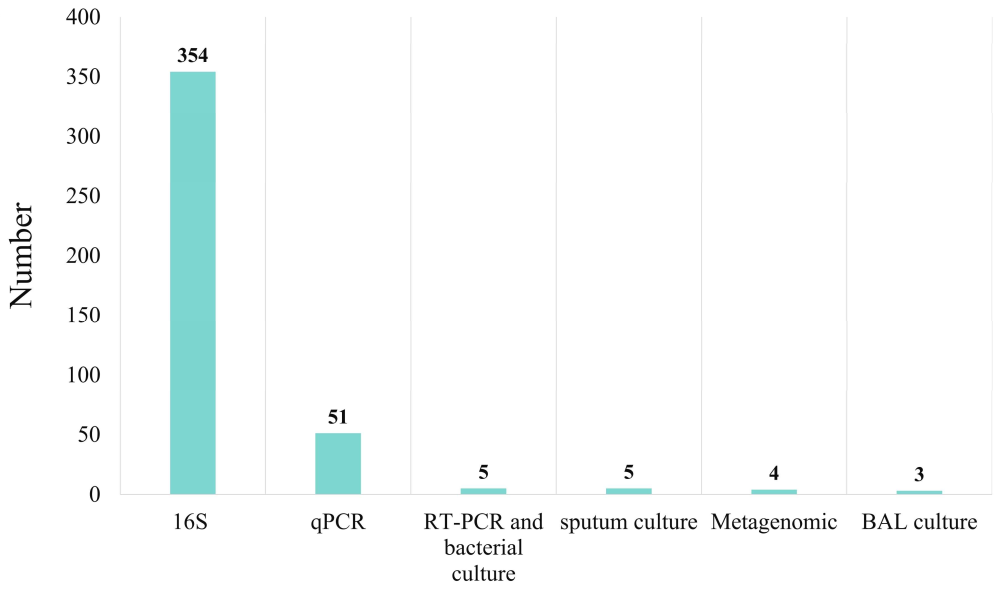

C

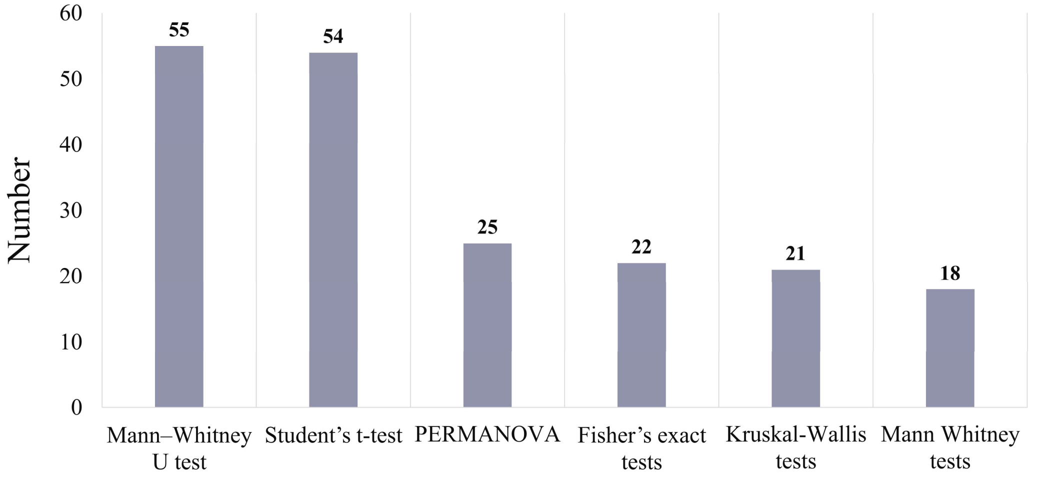

Figure S4. Overview of the data composition in LDMA. (A) The use of sequencing platforms (top 6). (B) The use of sequencing methods (top 6). (C) The use of statistical methods (top 6).
